# The final cut: cell polarity meets cytokinesis at the bud neck in *S. cerevisiae*

**DOI:** 10.1007/s00018-016-2220-3

**Published:** 2016-04-16

**Authors:** Maria Angeles Juanes, Simonetta Piatti

**Affiliations:** Centre de Recherche en Biologie Cellulaire de Montpellier, 1919 Route de Mende, 34293 Montpellier, France; Brandeis University, 415 South Street, Waltham, MA 02454 USA

**Keywords:** Cytokinesis, Budding yeast, Septins, Actomyosin ring, Mitotic exit network, Formins

## Abstract

Cell division is a fundamental but complex process that gives rise to two daughter cells. It includes an ordered set of events, altogether called “the cell cycle”, that culminate with cytokinesis, the final stage of mitosis leading to the physical separation of the two daughter cells. Symmetric cell division equally partitions cellular components between the two daughter cells, which are therefore identical to one another and often share the same fate. In many cases, however, cell division is asymmetrical and generates two daughter cells that differ in specific protein inheritance, cell size, or developmental potential. The budding yeast *Saccharomyces cerevisiae* has proven to be an excellent system to investigate the molecular mechanisms governing asymmetric cell division and cytokinesis. Budding yeast is highly polarized during the cell cycle and divides asymmetrically, producing two cells with distinct sizes and fates. Many components of the machinery establishing cell polarization during budding are relocalized to the division site (i.e., the bud neck) for cytokinesis. In this review we recapitulate how budding yeast cells undergo polarized processes at the bud neck for cell division.

## Introduction

Cell division is a fundamental but complex process that gives rise to two daughter cells. It includes an ordered set of events altogether called “the cell cycle” that culminates in cytokinesis, the final stage of mitosis leading to the physical separation of the two daughter cells. Symmetric cell division equally partitions cellular components between the two daughter cells, which are therefore identical to one another and often share the same fate. In many cases, however, cell division is asymmetrical and generates two daughter cells that differ in specific protein inheritance, cell size, or developmental potential [1–3]. An extensively studied example of asymmetric division is that adopted by stem cells, which give rise to one daughter cell that maintains its stemness and self-renewing potential while the other differentiates. The balance between self-renewal and differentiation is at the basis of tissue homeostasis and, not surprisingly, perturbing this delicate equilibrium can steer hyperproliferation and cancer [4–6].

**Fig. 1:**
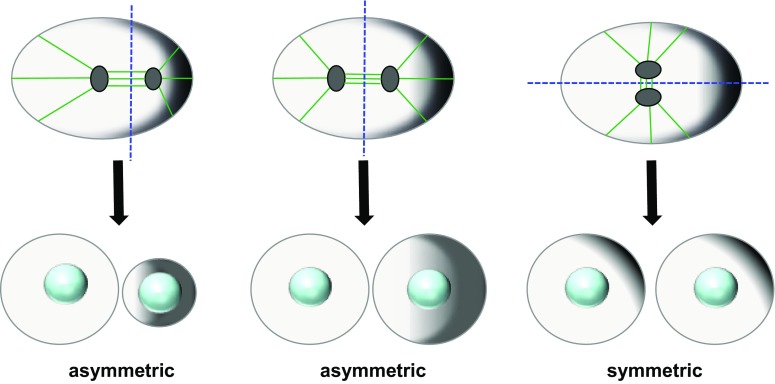
Spindle positioning relative to the polarity axis determines the outcome of cell division. The cartoon depicts a polarized cell, where a gradient of polarized factors increases from *left* to *right* (*gray shadow*). Depending on spindle positioning, which dictates the position of the cleavage furrow, cell division will be asymmetric or symmetric

Asymmetric cell divisions arise from special cellular architectures that make cells polarized, with a basal and apical side or a front and a rear. Cell polarity, however, is an intrinsic property of all types of cells and refers to spatial differences in shape, size and function of the cell. Depending on how the mitotic spindle and the cytokinetic furrow are positioned relative to the polarity axis the ensuing cell division is either symmetric or asymmetric [7] (Fig. 1).

Remarkably, positioning of the cytokinetic furrow is always coupled to spindle positioning (reviewed in [8]). Indeed, in many eukaryotic cells the mitotic spindle provides two non-mutually exclusive furrow-specifying signals [9, 10], one that originates from the spindle asters and the other from the central spindle, i.e., the region where polar microtubules interdigitate, which dictate positioning of the cleavage plane halfway between the two spindle poles (reviewed in [11]). Nonetheless, in some organisms, such as budding yeasts, where the site of cell division is defined early during the cell cycle and before spindle assembly, specific surveillance mechanisms delay the onset of cytokinesis until the spindle is properly positioned [12, 13]. Thus, cell polarity, spindle positioning and cytokinesis must be carefully orchestrated to ensure the successful physical separation of the two daughter cells, independently of whether cell division is symmetric or asymmetric.

The budding yeast *Saccharomyces cerevisiae* has proven to be an excellent system to investigate the molecular mechanisms governing cell polarity and cytokinesis. Budding yeast is highly polarized during the cell cycle and divides asymmetrically, producing two cells with distinct sizes and fates. Indeed, a bud emerges from the mother cell at the G1/S transition and keeps growing in size until cytokinesis, when it gives rise to a daughter cell. At this stage the mother cell is normally bigger than its daughter and progressively ages, while its daughter retains full lifespan [14]. Furthermore, mother and daughter cell undergo distinct transcriptional programs that allow, for instance, mating type switching to occur only in the mother cell, while expression of cell wall hydrolytic enzymes is restricted to the daughter cell [15, 16]. Strikingly, many components of the machinery establishing cell polarization during budding are relocalized to the bud neck (the constriction between mother and daughter cell where cytokinesis takes place) later on during the cell cycle for cytokinesis.

Besides these notable features, tractable genetics, powerful biochemistry, proteomics and cell biology approaches make yeast an attractive model for studying the intricate events governing asymmetric cell division, based on the precedent that fundamental principles in the control of cell division were discovered in budding yeast and proved fully applicable to higher eukaryotes.

In this review we recapitulate how budding yeast cells undergo polarized processes at the bud neck for cell division.

## Cell polarization

The ability to polarize is a fundamental property of all types of cells, being crucial for numerous cellular processes such as proliferation, differentiation and development. Simple unicellular eukaryotes, bacteria, cells of multicellular invertebrates or vertebrates are polarized. This results in an extraordinary diversity in the shapes of polarized cells that have been optimized for specialized cell functions, such as the ability to communicate over long distances (neurons), to provide barriers that regulate ion homeostasis between different biological compartments (epithelia), and to unevenly distribute cellular components to daughter cells upon cell division.

At first glance, this diversity of cell shapes and functions suggests that each cell type might have evolved completely different ways to generate cell polarity that distinguishes, for example, budding yeast from a multi-cellular epithelium. Surprisingly, while the final organization of polarized cells is diverse, the basic toolbox of proteins and core mechanisms responsible for polarization are conserved from yeast to humans [17]. Indeed, a common theme in the establishment of a site of polarization is the recruitment of specific lipids and proteins at a given position of the cell surface by membrane traffic along the cytoskeleton. Polarized distribution of macromolecules is achieved by delivery and fusion of vesicles with the plasma membrane (exocytosis), as well as by endocytic internalization and recycling of the molecules that diffuse laterally along the membrane. Signaling proteins, such as Rho-like GTPases (e.g. Cdc42 and Rho1) and Rab-like GTPases are then responsible for the reorganization of the cytoskeleton necessary to polarize the cell surface [18].

Defects in cell polarity can lead to cancer formation and metastasis. For instance, the ability of transformed epithelial cells to disseminate to distant organs is linked to a mesenchymal transition where their apico-basal polarity is lost [19, 20].

Since much of the cellular machinery that contributes to establishing and maintaining epithelial cell polarity is evolutionary conserved, dissecting polarity establishment in simple models, such as yeasts, has been invaluable to understand the basic principles of this process and its derangement during cancer progression.

### Polarized growth in the budding yeast *S. cerevisiae*

**Fig. 2:**
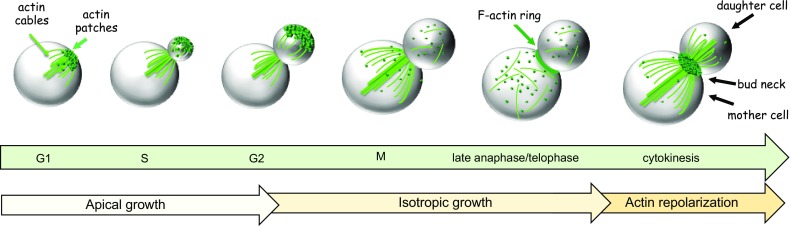
Organization of the actin cytoskeleton during budding yeast cell cycle. The cartoon illustrates budding yeast cells at different cell cycle stages and the distribution of actin structures (patches, cables and contractile F-ring) therein. Depending on whether actin organization is polarized, cell growth can be apical (directed towards the tip of the bud) or isotropic (with the bud expanding in all directions). After a transient depolarization of actin patches and cables in mitosis, the actin cytoskeleton repolarizes in telophase to bring about cytokinesis. See text for details

The budding yeast *S. cerevisiae* undergoes highly polarized cell growth throughout its life cycle and follows a stereotypical pattern of growth and division called budding [21, 22] (Fig. 2). Cells first select a site for bud emergence on the basis of cortical landmarks laid in relation to the previous division. Then, an axis of polarity directed toward this site is established by recruitment of signaling molecules. The established site then organizes a cytoskeletal framework targeting secretion for bud emergence. Further cell growth at this stage is mostly restricted to the bud, while the mother cell orchestrates the duplication and segregation of its organelles. Cells then undergo mitosis and cytokinesis, during which polarized secretion is directed to the bud neck to add new membrane and lay down the septum that separates mother and daughter cells.

Most aspects of polarized growth in *S.cerevisiae* arise from the polarization of the actin cytoskeleton [21–24]. Filamentous actin structures (F actin) comprise of (1) actin patches, (2) actin cables and (3) the cytokinetic actin ring.

1. Actin patches are mobile and discrete F actin rich bodies that are nucleated by the Arp2/3 complex and represent sites of endocytosis. Many endocytic proteins are indeed linked to the Arp2/3 for formation, maturation or scission of the actin patches (reviewed in [25–27]).
2. Actin cables are linear F actin bundles that act as “tracks” to guide the delivery of secretory vesicles towards the site of growth [28, 29]. They are anchored at discrete regions of the cell cortex (such as the nascent bud site or the bud neck) and radiate through the rest of the cell, underlying the cell cortex [28–30]. Actin cables are nucleated by the conserved diaphanous-related-formins (DRFs, below in detail) Bni1 (Bud-neck involved) and Bnr1 (*BNI1*-related) [31–33].
3. The cytokinetic F actin ring assembles at the bud neck, contracts and disassembles [34, 35]. This is closely followed by cell wall addition (i.e., septum formation) between the dividing cells. The two formins Bni1 and Bnr1 are collectively required for F actin ring assembly and contraction, with Bni1 playing a prominent role [34, 35].

As mentioned above, the very first step towards cytokinesis is the selection of an incipient bud site, which is chosen relative to cortical landmarks remaining from the previous cell division [36–38]. Excellent reviews can be found in the literature on this topic [23, 39–41], which therefore will not be treated here. Once the presumptive bud site has been selected in late G1, the actin cytoskeleton becomes highly polarized (Fig. 2). Cortical patches concentrate at the location of the new bud while actin cables emanate from this site. As a bud emerges, cortical patches initially cluster at its tip, while cables nucleated at the bud tip extend into the mother cell. This configuration supports trafficking required for bud growth from the tip (apical growth). Later on, patches and cables redistribute randomly within the bud, while cables in the mother cell still extend from the bud neck. During this phase, bud growth continues by expansion in all directions (isotropic growth). Finally, when cells exit mitosis actin repolarization occurs at the bud neck to support cytokinesis; the contractile F actin ring assembles and actin cables direct secretion towards the division site for septum formation (Fig. 2). Actin patches also concentrate at the mother and daughter side of the bud neck [28, 42], presumably for endocytic internalization and/or recycling of cytokinetic factors.

### Rho GTPases in the establishment of cell polarity

Rho GTPases are conserved proteins belonging to the Ras superfamily of small G proteins that are regarded as molecular switches, as they can oscillate between an inactive GDP-bound state and an active GTP-bound state [43, 44]. Like all G proteins, Rho GTPases are endowed with intrinsic GTPase activity. The rate-limiting step in GTPase activation is the release of GDP aided by guanine nucleotide exchange factors (GEFs), which allow GTPase-binding to GTP that in turn is present in the cells at higher concentrations than GDP. Conversely, GTP hydrolysis can be stimulated by GTPase-activating proteins GAPs) that shift the balance to the inactive state of the GTPase [45, 46]. Other regulators of Rho GTPases include the guanine nucleotide dissociation inhibitors (GDIs) that can lock the GTPase in the GDP- or GTP-bound form, as well as extract it from the membrane, thereby preventing its GEF-mediated activation [47].

Establishment of cell polarity in budding yeast requires the Rho GTPase Cdc42, which accumulates at the presumptive bud site through a process involving its GEF Cdc24 and the scaffold protein Bem1 that bridges Cdc24 to the Cdc42 effector Cla4, thereby generating a positive feedback loop that clusters Cdc42 to a single cortical site [48–50]. Cdc42 is further concentrated to a focused vertex by recycling mechanisms that include the GDI Rdi1 as well as the opposing activities of exo- and endocytosis [51, 52]. Once concentrated at a single focus, active Cdc42 organizes the actin cytoskeleton and septins to promote polarized secretion and cell growth (see below). Known Cdc42 effectors include the partially redundant PAK (p21-activated kinases) Cla4 and Ste20, which play major redundant roles in actin and septin organization [53–58], the formin Bni1 ([59], see below), the proteins Gic1 and Gic2, which promote septin recruitment and formin activity ([60–63], see below) and the Sec3 component of the exocyst complex, which plays essential role in exocytosis through vesicle targeting and docking to the plasma membrane [64, 65].

Besides Cdc42, budding yeast has five additional Rho GTPases that are named Rho1-5. Like Cdc42, Rho1 is essential for cell viability and plays a major role in cytokinesis through assembly of the cytokinetic contractile ring and the division septum (see below). Its effectors include formins [66, 67], the glucan synthase Fks1 [68], protein kinase C (Pkc1, [69, 70]) and the exocyst subunit Sec3 [71]. In contrast, Rho2-5 are dispensable for survival of yeast cells and their respective roles are ill-defined, although Rho3 and Rho4 share an essential role in the establishment of cell polarity and have been collectively implicated in formin activation [59, 66].

### Formins as key regulators of cell polarity and cytokinesis

Formins are large multi-domain proteins found in plants, fungi and mammals. Although their number is highly variable in different organisms, formin structure and function are highly conserved [72, 73].

**Fig. 3:**
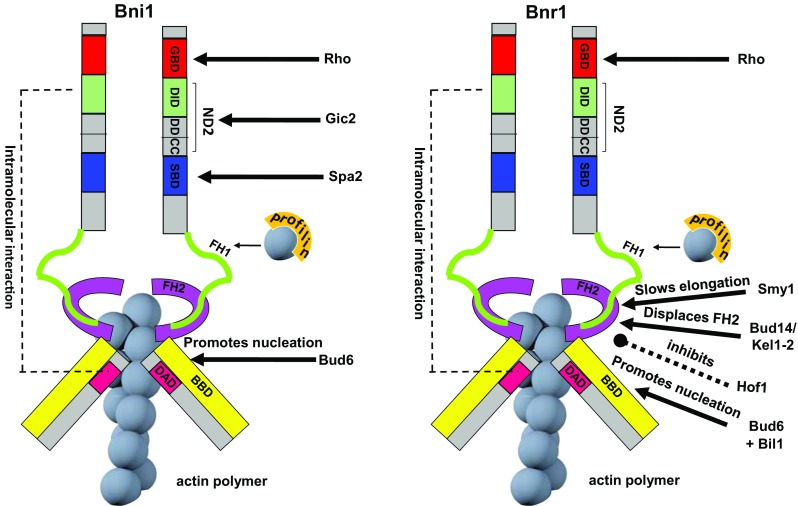
Structural organization of the yeast formins Bni1 and Bnr1. Formins form a doughnut-shaped dimer that encircles the nascent actin filament during its elongation. The main interactors and regulators of each formin are depicted. See text for details. *GBD* GTPase-binding domain, *DID* diaphanous inhibitory domain, *DD* dimerization domain, *CC* coiled coil, *SBD* Spa2-binding domain, *FH1/2* formin homology domain, *DAD* diaphanous auto-regulatory domain, *BBD* Bud6-binding domain

In budding yeast there are two arrays of actin cables, one polarized toward the bud cortex and the other toward the mother-bud neck, that are nucleated by the two formins, Bni1 and Bnr1 [31, 32, 67, 74–76]. In budding yeast, neither one of the two formins is essential, but cell viability requires at least one of them [66], suggesting that they share at least one essential function. However, Bni1 and Bnr1 clearly also play distinct cellular roles, which is highlighted by their different patterns of cellular localization and respective mutant phenotypes [77–80]. From bud emergence to mitotic exit Bnr1 resides at the bud neck, where it is relatively static and nucleates actin cables extending into the mother cell [76, 77, 81]. In contrast, Bni1 localizes throughout most of the cell cycle to the bud tip, where it nucleates actin cables, and to the bud neck immediately before cytokinesis, when it replaces Bnr1, helping to form the contractile actomyosin ring (CAR) [77, 82]. At the bud tip, Bni1 binds to several components of the polarisome (i.e., Spa2, Pea2 and Bud6, [31, 83]), a protein complex involved in cell polarity that localizes at sites of polarized growth [84]. Two motifs named SBD (Spa2-binding domain) and BBD (Bud6-binding domain) (Fig. 3) have been mapped in the middle and C terminal region of Bni1, respectively, linking Bni1 to the polarisome [78, 79, 83]. The Spa2–Bni1 binding is required for Bni1 localization at the bud tip and for proper regulation of actin architecture. Indeed, in the absence of Spa2, Bni1 gets redistributed to the cytosol instead of localizing at bud cortical sites [83]. In contrast, loss of Bud6 has a minor impact on Bni1 localization [31]. However, a C terminal fragment of Bud6 can stimulate the actin-polymerizing activity of Bni1, as supported by in vitro experiments that led to the proposal that Bud6 acts as a nucleation-promoting factor [79, 85]. Interestingly, Bud6 can also enhance in vitro actin nucleation by the other formin Bnr1 when assisted by the Bil1 protein (Bud6-interacting ligand) [86], in agreement with previous data showing that the C terminal part of Bud6 participates in formin-dependent actin cable organization in vivo [78].

Formin recruitment to and release from membranes, as well as its actin-nucleating activity, involve additional formin modifications and/or binding partners that tune their function. The formin homology domain 1 (FH1, Fig. 3) is a proline-rich motif that binds profilin, among other proteins. Profilin is an actin-binding protein that recruits actin monomers to the active region of formin when bound to FH1 [66, 74], thereby stimulating formin-induced actin polymerization [32, 87]. The second formin homology domain (FH2, Fig. 3) lies next to FH1 and is required to form a doughnut-shaped formin dimer that encircles the nascent actin filament during its elongation [88]. Consistently, the dimeric architecture of formin is relevant for its actin nucleation activity [87, 89].

Formins have two additional important regulatory motifs, the DID (Diaphanous Inhibitory Domain) and the DAD (Diaphanous Auto-regulatory Domain) (Fig. 3) that were originally identified in the *Drosophila* formin Diaphanous [90]. DID and DAD can interact with one another within the same formin molecule, thereby locking formin in a close inhibited state [91, 92]. Other formin domains can relieve this autoinhibition to promote an open and active state. Among them, the GBD (GTPase binding) and the ND2 (N-terminal) domains (Fig. 3) bind to Rho GTPases and the Gic2 protein, respectively, and also control Bni1 localization at the bud tip [62, 93–95]. The GBD is located next to the DID domain, and its binding to Rho relieves formin autoinhibition by hindering DID interaction with DAD [94, 96–100]. Whether formin binding to a Rho GTPase is always necessary for its activation is unclear, especially since other domains can also relieve autoinhibition.

To add additional layers of complexity to formin regulation, other cofactors modulate the biological function of formins. Tropomyosins, which are master regulators of muscle cells contraction, also play key roles in non-muscle cells by controlling actin dynamics and cell migration [101, 102]. In budding yeast, they control assembly and stability of actin cables [103]. Additionally, tropomyosins promote formin-mediated formation of contractile ring assembly in fission yeast [104], raising the possibility that they might play a similar function also in budding yeast.

Yet another formin regulator is the kinesin-like myosin-passenger protein Smy1, which acts as a Bnr1 damper in vitro and in vivo without affecting Bni1 [105, 106]. Smy1 slows down the elongation rate of Bnr1-mediated actin polymerization by direct binding to Bnr1. Accordingly, cells lacking Smy1 show extremely long actin cables with prominent defects in their architecture [105]. Recently, Smy1, Bnr1 and the myosin V motor protein Myo2 that delivers Smy1 to formin have been involved in an “antenna mechanism” that senses and controls the length of actin cables [107].

Another set of formin tuners act in parallel with the ones listed above to ensure proper actin cable architecture. A complex formed by Bud14 and the Kelch-domain proteins Kel1 and Kel2, which are involved in cell polarity and morphogenesis, senses the length of actin cables and eventually displaces Bnr1 from actin filaments [108–110]. Bud14 does not suppress Bnr1 actin-polymerizing activity but rather the permanence of formin on actin, thereby attenuating the elongation rate of actin filaments.

The F-BAR protein Hof1, which controls septin organization and septum deposition ([81, 111, 112], see below), inhibits the actin-nucleating activity of Bnr1 both in vitro and in vivo [113], thereby tuning the architecture of the actin cable network. Conversely, Bnr1 is somehow activated in vivo by septins (see below) and the septin-associated kinase Gin4 [114].

Finally, formin activity is likely controlled by post-translational modifications. For instance, phosphorylation of Bni1 by the Prk1 kinase unleashes its autoinhibition [115]. In mating cells, Bni1 phosphorylation by the Fus3 MAP kinase is important for its localization to and assembly of actin cables [116], while dephosphorylation of both Bni1 and Bnr1 by Cdc14, as well as Bnr1 dephosphorylation by protein phosphatase 1 (the PP1 Glc7), seems to trigger the replacement of Bnr1 with Bni1 during mitotic exit [117, 118]. Finally, a truncated variant of Bni1 was recently shown to be ubiquitylated in vivo by the Rsp5 E3-ubiquitin ligase and subsequently degraded to reorganize the actin cytoskeleton under stress conditions and wound healing [119].

### Actin polarization at the bud neck for cytokinesis

Just before cell division, the actin cytoskeleton repolarizes to the bud neck (Fig. 2). Thus, actin structures (actin cables, actin patches and the F actin ring) converge at the cell division site. In particular, actin cables rearrange to be polarized towards the bud neck and ensure that membrane trafficking will bring secretory vesicles and proteins to the cytokinesis site to bring about membrane closure. This remarkable reorganization of the actin cytoskeleton is driven by inactivation of mitotic cyclin B-CDK complexes at mitotic exit [42, 120] (see “The mitotic exit network”). Approximately at the same time, many polarity factors, such as the Cdc42 and Rho1 GTPases, polarisome components (e.g. Bni1, Spa2 and Bud6) and the exocyst complex, translocate from the bud tip to the bud neck, thus contributing to the actin rearrangements accompanying this transition [76, 81, 82, 84, 121–123]. Most likely, the relocalization of some of these proteins occurs to reinforce Bni1-dependent polymerization of actin cables and ring at the bud neck. While Rho1 presumably directly promotes local Bni1 activation for F actin ring assembly (see below, [124]), it has not been established if Cdc42 directly activates Bni1 for actin polymerization prior to cytokinesis. However, inactivation of the redundant PAK kinases Ste20 and Cla4, which are known Cdc42 effectors, during mitosis abolishes actin repolarization at the bud neck [55], suggesting that Cdc42 might have an indirect role in formin activation at cytokinesis. In turn, Ste20, and perhaps Cla4, might regulate Bni1 activity through direct phosphorylation [54, 125]. Interestingly, although Cdc42 persists at the bud neck until cytokinesis has been accomplished, the levels of active GTP-bound Cdc42 decrease at cytokinesis [123, 126, 127]. Inhibition of Cdc42, and Ste20 downstream to Cdc42, is in turn important for efficient recruitment of cytokinesis factors to the bud neck and proper cell division [128]. Consistent with the antagonism between Cdc42 and Rho1/RhoA that has been shown in many eukaryotic systems, the polo kinase Cdc5 is required for Cdc42 inhibition while promoting the recruitment of Rho1 to the neck through phosphorylation of one of its guanine nucleotide exchange factors (GEFs) [129, 130].

While Spa2 mediates localization of the formin Bni1 at the bud tip [83], it is dispensable for its redistribution to the bud neck at cytokinesis [82]. However, Spa2 and Bud6 might contribute to formin-dependent actin polymerization. Although the precise mechanism by which Bni1 relocates from the bud tip to the neck is still elusive, the Cdc14 phosphatase and Bni1 phosphorylation were shown to be involved in this process [117]. A good candidate for promoting Bni1 recruitment to the bud neck is the exocyst complex. The exocyst is a conserved protein complex made by eight subunits (Sec3, Sec5, Sec6, Sec8, Sec10, Sec15, Exo70 and Exo84) that tethers exocytic vesicles to the plasma membrane during secretion [131–133]. Inactivation of the exocyst complex through temperature-sensitive mutations leads to disappearance of actin cables and a general depolarization of actin, which suggests that the exocyst regulates actin dynamics [134, 135]. Strikingly, in fission yeast Sec3 is essential for the localization of the formin For3 (the fission yeast counterpart of Bni1) at the plasma membrane where the two proteins interact [136]. Importantly, the distribution and/or activity of the exocyst complex is controlled by both Cdc42 [64, 137, 138] and Rho1 [71], presumably in a reciprocal manner depending on the cell cycle stage, suggesting yet another possible route through which Rho-like GTPases might control formin activity.

## The septin ring

### The septin ring and cytokinesis

Studies in budding yeast and mammalian cells indicate that septins act as scaffold to recruit cytokinetic factors to the site of cell division (reviewed in [139, 140]). Septins were first discovered in budding yeast through a genetic screen for mutants defective in cell division [141] and are cytoskeletal GTP-binding proteins that form oligomeric complexes that can in turn self-organize in higher-order structures, such as filaments and rings. Although septins have been found at the division site in most cell types examined so far, the extent to which they contribute to cytokinesis varies from one organism to another. For example, in the fission yeast *S. pombe* septins appear at the division plane only after the cytokinetic ring has fully assembled [142, 143] and their deletion causes only a mild cell separation defect [144, 145]. In stark contrast, several of *S. cerevisiae* septins are essential for viability and cytokinesis.

The five septins expressed in vegetative budding yeast cells (Cdc3, Cdc10, Cdc11, Cdc12 and Shs1) form hetero-octamers composed by two copies of the core Cdc10, Cdc3 and Cdc12 subunits and two copies of the alternative septins Cdc11 and Shs1 arranged in palindromic linear rods [57, 146, 147]. The rods collide on the plasma membrane to join end-to-end in non-polar filaments [148] that in turn organize in a ring. Recent data showed that the non-essential yeast septin, Shs1, curves septin filament bundles into rings in vitro and promotes proper septin organization at the bud neck in vivo [147].

The bud neck protein Bni5 had been identified as multicopy suppressor of septin mutants [149] and has been recently shown to crosslink septin filaments in vitro [150], likely providing structural stability to the septin ring in vivo. Bni5 directly interacts with the septins Cdc11 and Shs1, as well as with Myo1, mediating its recruitment to the division site throughout most of the cell cycle until cytokinesis [151–153].

Septins associate with membranes, and in particular with positively charged phosphoinositides, such as phosphatidylinositol-4,5-diphosphate (PIP2) [154], through a highly conserved polybasic region at the N terminus [155, 156]. In budding yeast PIP_2_ is enriched in membrane areas of polarized growth and the bud neck [157] and it stimulates formation and organization of septin filaments that are in turn essential for cell viability [154, 158].

**Fig. 4:**
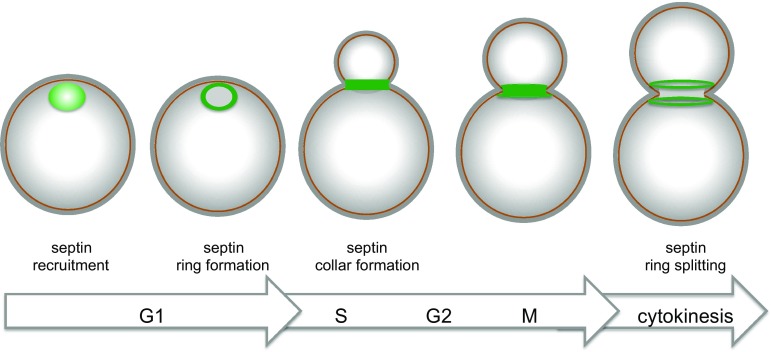
Main steps in budding yeast cytokinesis. See text for details

Septins are first recruited to the presumptive bud site as unorganized septin clouds or patches, which are then rapidly transformed into a cortical septin ring in late G1. Fluorescence Recovery After Photobleaching (FRAP) experiments indicate that septin structures prior to bud emergence are highly dynamic. At the time of bud emergence the septin ring expands into a rigid hourglass-like structure referred to as septin collar, which spans the whole bud neck and scaffolds many cytokinetic factors [159, 160]. Immediately prior to cytokinesis the collar splits into two distinct rings that sandwich the contractile actomyosin ring (CAR, see below) and are highly dynamic (reviewed in [139, 140]) (Fig. 4). The physiological relevance of ring splitting for cytokinesis has yet to be elucidated. However, coincident with or immediately after septin-ring splitting the CAR constricts in between the split septin rings and the cleavage furrow ingress, bringing about deposition of the primary septum [161, 162]. Remarkably, septin ring splitting is accompanied by a striking change in septin arrangement that was revealed by polarized fluorescence microscopy. Indeed, while septin filaments inside the collar are arranged in parallel arrays aligned along the mother-bud axis, they are found rotated by 90° in split septin rings [163, 164]. How these observations can be reconciled with earlier electron microscopy (EM) studies showing that septin rings are made by circumferential septin filaments encircling the bud neck [165] has been subjected to extensive debate. Recent data obtained by platinum-replica EM and correlative light/EM suggest that the early septin collar is made by double septin filaments oriented along the mother-bud axis, while later on during mitosis it acquires orthogonally oriented circumferential septin filaments that confer a gauze-like appearance to the structure [166]. During septin ring splitting the septin double filaments are somehow depolymerized, leaving two parallel rings of filaments around the bud neck. Interestingly, although the myosin II Myo1 and the non-essential septin Shs1 are not strictly required for septin collar formation, they seem to affect the overall organization of the mature septin collar [166].

The Cdc42 GTPase is essential for septin recruitment to the presumptive bud site [63, 167] and cycles of Cdc42 GTP-binding and hydrolysis are required for septin collar formation [159, 168]. Among the known Cdc42 effectors, the paralogous membrane proteins Gic1 and Gic2 seem to play a crucial role in septin recruitment. Accordingly, septin deposition and budding mostly fail in *gic1 gic2* double mutants at high temperatures [63]. Gic1 localizes at the presumptive bud site and bud tip at early stages of the cell cycle and at the bud neck later on [63], and it has been recently shown to bundle and crosslink septin filaments in vitro, thereby stabilizing them [169]. Surprisingly, the inactive GDP-bound form of Cdc42 was found to bind directly septin filaments and to depolymerize them when present at high concentrations, while creating lateral crosslinks between septin filaments at low concentrations. On the basis of these and other observations it has been proposed that the initial recruitment of septin octamers to the future bud site is promoted by Cdc42-GDP itself; afterwards, septin filaments get bundled and stabilized by Gic1, presumably bound to Cdc42-GTP [169]. Once a polarized cap of septin filaments has been formed at the presumptive bud site, the septin ring is sculpted by polarized exocytosis that creates a hole in the middle of the cap [170]. At cytokinesis, Cdc42 accumulates at the bud neck where it might induce septin depolymerisation [169], to favor recycling of septin octamers for the following cell cycle [171]. Intriguingly, septins inhibit Cdc42 activity at the bud neck in a negative feedback loop through GTPase-activating proteins, which promote the Cdc42 GDP-bound inactive state [170]. Of note, according to the model proposed by Sadian et al. [169] this event could stimulate, at the same time, further recruitment of septin octamers.

Direct binding to septin filaments and activation of Gic1 and Gic2 are not the only function of Cdc42 in septin regulation. Another Cdc42 effector, the PAK kinase Cla4, directly phosphorylates the septin Cdc10 in vitro and in vivo, thereby preserving the integrity of septin architecture [57]. Furthermore, the formin Bni1, which is another effector of Cdc42 (see above), contributes to septin ring formation along with Cla4 [56].

Anillin is a multi-domain cytokinesis protein that in metazoans interacts with a plethora of partners, including actin, myosin, septins and formins, among many others (reviewed in [172]). In budding yeast, the anillin-like protein Bud4 associates with septins in mitosis and colocalizes with the septin ring until after the next G1 phase, to then disappear at the onset of budding [173, 174]. Bud4 is required to stabilize the septin ring during splitting [162, 175], similar to the fission yeast anillin Mid2 [142, 143]. However, the mechanism underlying this stabilization has yet to be discovered, as well as the physiological relevance of septin ring splitting. Indeed, *BUD4* deletion causes mild cytokinesis defects that get more pronounced in sensitized mutant backgrounds [176].

The Rho1 GTPase, besides playing a pivotal role in CAR assembly [124](see below), has been recently shown to stabilize septins during their recruitment to the presumptive bud site through activation of its effector protein kinase C (Pkc1). Pkc1 in turn modulates the turnover at the bud neck of the F-BAR protein Syp1 through direct phosphorylation [177]. Syp1 is an endocytic protein that has been implicated in timely septin deposition and in stimulating septin ring dynamics through an unknown mechanism [177, 178]. Remarkably, Syp1 is recruited to the presumptive bud site in G1, at the same time as septins, and forms a ring that surrounds and is larger than the septin ring. After budding the Syp1 ring is thus found asymmetrically located on the mother side of the septin collar [177]. This peculiar spatial arrangement of Syp1 relative to septins is highly reminiscent of the coordination between secretion and endocytosis occurring during the establishment of cell polarity in G1, where endocytosis corrals at the membrane a vertex of active exocytosis for bud emergence [51]. Thus, altogether these data raise the possibility that timely accumulation of septins at the future bud site in late G1 might be facilitated by endocytic recycling. Interestingly, the EH domain-containing protein Ede1, which is a major partner of Syp1 for endocytosis [179, 180], has been recently implicated in cytokinesis [181].

### Other functions of the yeast septin ring

Besides being necessary for cytokinesis, the budding yeast septin ring has been implicated in several other polarized processes. Some of them are intimately linked to the second main function of septins, in addition to scaffolding, as cortical barriers to prevent the free diffusion of membrane proteins between different compartments (reviewed in [182, 183]). For instance, the septin ring hampers the diffusion to the mother cell of the mitotic exit regulator Lte1, normally localized in the bud cortex, thereby ensuring the proper coupling between correct spindle positioning and mitotic exit [184]. The septin ring also restricts to the bud the accumulation of the machinery responsible for the asymmetric localization of certain mRNAs [185, 186]. Additionally, the septin ring segregates some membrane proteins of the endoplasmic reticulum (ER) to a specific cell compartment, without influencing the distribution of ER luminal proteins that instead remain freely diffusible [187]. Finally, the septin ring contributes to proper spindle positioning early in mitosis [188] and promotes mitotic entry by scaffolding at the bud neck the machinery responsible for the degradation of the Wee1-like kinase Swe1, which inactivates mitotic cyclin-dependent kinases (CDKs) by inhibitory phosphorylation [189–192].

### Septin post-translational modifications

Septins are targeted by many post-translational modifications, such as phosphorylation, acetylation, sumoylation and ubiquitination that likely modulate the state transitions of the septin ring during the cell cycle. Several protein kinases, such as Cla4, the Nim (never in mitosis)-related kinases Gin4, Kcc4 and Hsl1, as well as the Elm1 kinase, localize at the bud neck in a septin-dependent manner and promote septin collar formation and stabilization (reviewed in [139, 140, 193]). Specifically, Cla4 phosphorylates several septins in vitro [57] and is regulated by Elm1 [194], while Gin4 phosphorylates Shs1. Gin4 is phosphorylated and activated by Elm1, which together with Cla4 also promotes its recruitment to septins [190, 195, 196]. G1 cyclin-dependent kinases (CDKs) have also been implicated in septin phosphorylation, and CDK-dependent phosphorylation of Shs1 was shown to enhance its interaction with Gin4 [197, 198]. Although the extensive level of phosphorylation and the large number of kinases involved have hampered so far the functional dissection of this post-translational modification, as well as the assessment of the precise role of the Hsl1 and Kcc4 kinases in septin regulation, altogether septin phosphorylation seems to accompany septin ring stabilization. Consistently, the protein phosphatase PP2A bound to the Rts1 regulatory subunit reverses phosphorylation of at least Shs1 and contributes to timely septin disassembly after cytokinesis [199].

Several septins were found to be sumoylated in mitosis by the Siz1 and Siz2 Sumo-ligases [200, 201]. A mutant lacking the major sumoylation sites in Cdc3, Cdc11 and Shs1 displays prominent defects in septin ring disassembly at the end of mitosis. However, the lack of a similar phenotype in *siz1 siz2* double mutants or in *ubc9* temperature-sensitive mutant that affects the only Sumo-conjugating enzyme leaves open the possibility that the sumoylated lysines of septins might be targeted by other post-translational modifications, namely acetylation or ubiquitination [201].

The septins Cdc11 and Shs1 have been recently shown to be ubiquitinated by the Dma1 and Dma2 E3 ubiquitin ligases [202], which had been previously involved in septin ring stability [203, 204]. However, the role of septin ubiquitination remains to be established.

Finally, septin acetylation by the NuA4 and Esa1 lysine acetyltransferases was found to stabilize the septin collar [205]. Intriguingly, in mutants affecting septin acetylation septin complexes contain actin, suggesting that interaction of septins with the actin cytoskeleton might be deleterious for septin collar stability. Along the same line, in mammalian interphase cells, where septins colocalize with actin in long linear bundles or arcs, actin depolymerisation by cytochalasin D treatment leads to the formation of septin rings [206].

Although septin filaments are apolar and the septin collar is symmetric [207], several proteins that are recruited to the bud neck in a septin-dependent manner localize asymmetrically on the septin collar. For instance, the PP1 phosphatase Glc7 associated to its regulatory subunit Bni4, the F-BAR protein Syp1, the kinase Gin4 and the sumo-ligases Siz1 and Siz2 localize on the mother side of the septin collar, while the kinase Kcc4 is restricted to its bud side [201, 208, 209]. How this asymmetry is generated is unclear but it might rely on specific posttranslational modifications and in turn generate asymmetric septin modifications.

## Assembly of the actomyosin ring

In many eukaryotic organisms, including budding yeast, cytokinesis involves a contractile actomyosin ring (CAR) made by the motor protein myosin II and actin filaments. The CAR assembles at the site of cell division and drives furrow ingression (reviewed by [210]). In *S. cerevisiae* the CAR is coordinated with and guides the formation of the primary septum, which involves the addition of a new cell wall between the dividing cells (see “Constriction of the actomyosin ring”). In the absence of a functional CAR budding yeast cells fail to invaginate the plasma membrane during cytokinesis but in some strain backgrounds they can eventually survive thanks to the formation of aberrant remedial septa [34, 211–213].

CAR assembly in budding yeast is a sequential order of events that starts in late G1 with the septin-dependent recruitment to the presumptive bud site of the single myosin type II heavy chain Myo1, along with its regulatory light chain Mlc2, [214, 215]. In mitosis, at a time when the essential myosin light chain Mlc1 appears at the bud neck, Myo1 interacts also with Mlc1. In telophase Mlc1 promotes the accumulation to the bud neck of the IQGAP protein Iqg1, which is in turn essential for recruiting filamentous actin (F actin) to the CAR, as well as for a second wave of Myo1 targeting to the neck that further increases its local levels [151, 214, 216–220].

The two formins Bni1 and Bnr1 are also essential for the engagement of F actin at the CAR [112, 124], although their exact relationship with Iqg1 has not been established. Furthermore, formins have been recently shown to contribute to the accumulation of Mlc1 at the bud neck during cytokinesis [221], suggesting an additional mechanism by which they could participate in CAR assembly. In principle, many of the formin regulators described above (see “Rho GTPases in the establishment of cell polarity”) could contribute to timely CAR assembly to various extents.

In *S. pombe* recruitment of formins to the medial cortex, where cytokinesis occurs, is partly mediated by the cytokinetic protein Cdc15, which contains an F-BAR domain (F-BAR: ‘FCH and BAR’, where FCH = Fes/CIP4 Homology and BAR = Bin-Amphiphysin-Rvs) to bind membranes and is a key regulator of CAR assembly and stability [222–226]. Its counterpart in budding yeast, called Hof1 (homologue of fifteen), interacts and partially constricts with the CAR but is thought to be dispensable for CAR function. Rather, it has been implicated in primary septum formation (see below) [81, 111, 112, 227, 228]. However, it has been recently reported that Hof1 functions redundantly with the yeast amphiphysin Rvs167, which also contains a BAR domain, in promoting F actin assembly at the CAR [229], suggesting that it might have a conserved role in CAR formation.

The precise function of the Mlc2/Myo1 complex early in the cell cycle is unknown, but has been proposed to stimulate the retrograde flow of cargos on actin cables [230]. In late G1 Myo1 recruitment to the neck depends on septins and the septin-binding protein Bni5 (see below) and is characterized by high turnover [34, 149, 152, 231]. As the cell cycle proceeds, Myo1 remains localized at the bud neck until F actin is recruited around anaphase to form the CAR [34, 35]. Shortly before cytokinesis Myo1 levels further increase at the bud neck through a mechanism that involves Mlc1 and Iqg1, and Myo1 becomes immobile at the neck where it acts as scaffold for the cytokinetic machinery [151, 231].

As already mentioned, the CAR consists of actin filaments nucleated by formins, which are in turn activated by the Rho1 GTPase [67, 112, 124]. Indeed, Rho1 is essential for assembly of the F actin ring [124], similar to RhoA in many eukaryotic organisms (reviewed in [232]). Rho1 is recruited to the division site through a major mechanism involving its GEFs (Rom1, Rom2 and Tus1) and a distinct backup mechanism depending on interaction between the C terminus of Rho1 and acidic phospholipids [129]. Furthermore, the polo kinase Cdc5 is necessary for Rho1 and Bni1 localization at the bud neck, probably through direct phosphorylation of the GEFs Rom2 and Tus1 [130].

The exact arrangement of actin filaments inside the ring and the precise mechanism by which budding yeast formins contribute to the assembly/contraction of the cytokinetic ring have not been fully elucidated. In fission yeast it has been suggested that pre-existing actin cables might coalesce into the cytokinetic actin ring [233]. However, this model does not seem to apply to budding yeast, where cells with F actin rings are mostly devoid of actin cables, suggesting that the two structures compete with one another for formin-dependent polymerization [124].

Likely, both formins Bnr1 and Bni1 can promote the assembly of the actin ring at the budding yeast bud neck since both form actin cables and localize at the bud neck, albeit in a mutually exclusive manner, during CAR assembly. Consistently, lack of either formin does not affect CAR formation, whereas inactivation of both, as well as inactivation of tropomyosins and profilin, disrupts actin recruitment to the CAR [112, 124].

Strikingly, at the onset of cytokinesis, concomitant with CAR contraction, Bnr1 leaves the bud neck through a process that appears to be linked to its dephosphorylation [117, 118], thus empowering Bni1 as a prominent player for CAR contraction. Accordingly, in *bni1* null mutant cells the actin ring still forms but often fails to contract, while this is not the case for *bnr1∆* mutants [112]. Thus, although Bni1 and Bnr1 seem to play overlapping roles in actin ring formation, their coordinated interplay is important for proper ring contraction, possibly through the interaction with other polarized factors involved in cytokinesis.

The exact function of the IQGAP Iqg1 in CAR assembly has not been fully understood. Mammalian IQGAP can crosslink actin filaments [234] and has been proposed to act as scaffold for the actin assembly machinery [235, 236], while budding yeast Iqg1 can bind actin through its N terminal calponin-homology domain (CHD), suggesting that it might directly recruit actin to the CAR [217, 218]. Consistently, *IQG1* overexpression causes premature actin ring formation [217]. How Iqg1 and formins cooperate to assemble the actin ring is unclear. Mammalian IQGAP interacts physically with the formin Dia1 and is required for its proper localization [237]. Similarly, *C. albicans* Iqg1 associates with both formins Bni1 and Bnr1 and promotes efficient recruitment of Bni1 to the bud neck [238]. Thus, Iqg1 might on one side organize actin filaments polymerized by formins and on the other favor efficient formin activity.

## Constriction of the actomyosin ring

**Fig. 5:**
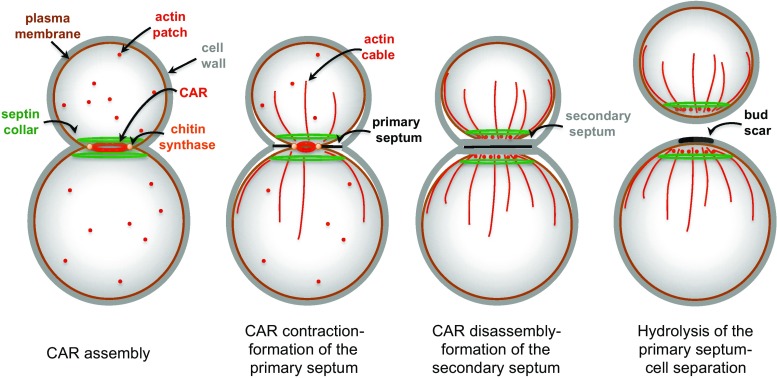
The septin ring during budding yeast cell cycle. Sequential stages of septin organization during the cell cycle of budding yeast

Shortly after its complete formation the CAR constricts (Fig. 5). In many organisms, CAR constriction during cytokinesis is thought to drive invagination of the overlying plasma membrane inward generating the force to cleave the cell in two (reviewed by [210]). In budding yeast CAR also drives membrane deposition through vesicle targeting and contributes to formation of the primary septum. The mechanism of CAR constriction has originally been inferred from that by which actomyosin generates force in the striated muscle, which stems from the sliding of bipolar myosin filaments along actin filaments that are organized in regular antiparallel arrays [239, 240]. However, this model does not seem to apply to budding yeast CAR. Indeed, Myo1 levels progressively decrease as the CAR constricts [162, 241], while they would be expected to remain constant if a sliding mechanism fully accounted for constriction. Furthermore, unlike in other organisms, the motor domain of Myo1 is not strictly required for CAR constriction and cytokinesis [151, 242, 243], while the rest of the protein is essential in most strain backgrounds and necessary for actin assembly in the CAR [34, 213, 244]. In agreement with these observations, CAR contraction in *S. cerevisiae* has been recently shown to be mainly driven by actin depolymerisation promoted by the cofilin Cof1. Actin depolymerisation by Cof1 synergizes with the motor activity of Myo1 to promote fast CAR constriction, with the latter mechanism playing a less prominent role than the former [243]. Since the action of cofilin can be stimulated by actin crosslinking to generate contractile stress [245], the IQGAP Iqg1 has been proposed to play such a role during yeast cytokinesis [243]. Consistently, deletion of the C terminal GTPase-activating protein-related domain of Iqg1 prevents CAR constriction without affecting CAR assembly [218], while the phosphorylation-deficient mutants of *IQG1* slow down CAR constriction, while advancing CAR formation [238, 246]. Interestingly, although Iqg1 is necessary for CAR assembly and constriction, its ubiquitin-dependent degradation mediated by the anaphase-promoting complex is important for CAR disassembly after cytokinesis [241].

Around the time of CAR constriction the myosin V Myo2, which transports post-Golgi vesicles along actin cables, and the exocyst complex, which tethers secretory vesicles to the plasma membrane, get recruited to the CAR to promote delivery of membrane and essential cargoes to the division site [120, 151, 162, 199, 231]. The essential myosin light chain Mlc1 is required for Myo2 tethering to the bud neck [247], thereby coordinating CAR formation with membrane trafficking. Remarkably, interfering with membrane traffic at the bud neck through mutations affecting Myo2 or exocyst subunits leads to CAR destabilization during constriction, without affecting CAR assembly [120]. Similarly, loss of the chitin synthase Chs2, which is required for primary septum formation and is transported to the bud neck through Myo2- and exocyst-mediated secretion (see below), affects the stability of the CAR during contraction [120, 212], suggesting that CAR constriction, secretion and septation are intimately connected processes (Fig. 5).

## Septum formation

Contractile actomyosin ring constriction in budding yeast is coupled to the centripetal deposit of a primary septum that physically separates the two daughter cells. The primary septum is a chitin disk deposited by the action of the chitin synthase 2 (Chs2, Fig. 5). Chs2 is synthesized in G2/M and accumulates in the endoplasmic reticulum (ER) until the end of mitosis [248]. Inactivation of mitotic CDKs or Chs2 dephosphorylation by the Cdc14 phosphatase triggers the translocation of the chitin synthase Chs2 from the ER to the bud neck [249–251] (see also “The mitotic exit network”). As mentioned above, Chs2 is a cargo of the exocyst complex and delivered to the bud neck through Myo2-dependent transport along actin cables. Then, Chs2 persists at the bud neck to form the primary septum in coordination with CAR contraction [120, 212, 248] (Fig. 5). Once this process is accomplished, Chs2 is removed from the neck by endocytosis and transferred to the vacuole for degradation [252].

The F-BAR protein Hof1 forms a ternary complex with the cytokinetic proteins Inn1 and Cyk3 to couple CAR contraction with membrane ingression and primary septum deposition. Mutants affecting the Hof1–Inn1–Cyk3 complex exhibit various degrees of cytokinesis defects and Inn1, but not Hof1 and Cyk3, is essential for cell viability [111, 112, 227, 253–257]. Furthermore, overexpression of *HOF1* and *CYK3* efficiently rescues the cytokinetic defects of *iqg1* mutant cells without restoring a CAR [254]. The Hof1–Inn1–Cyk3 complex is thought to be mainly involved in cytokinesis by promoting primary septum formation, most likely by activating the chitin synthase Chs2 [181, 251, 255, 258]. Consistently, Hof1 interacts physically with Chs2 and stabilizes it at the bud neck during CAR constriction, while overexpression of *HOF1* or *CYK3* rescues the cytokinetic defects of hypomorphic, but not null, *chs2* mutants [111, 256]. In addition, mutants affecting the Hof1–Inn1–Cyk3 complex fail to undergo centripetal and symmetric CAR contraction, resulting in CAR destabilization during constriction similar to *chs2* mutant cells [120, 212, 227, 228, 257].

Shortly after the primary septum starts being assembled, cells deposit a secondary septum composed of glucans (polymers of glucose) and mannoproteins (heavily glycosylated cell wall proteins bearing abundant mannose sugars) on each side of the chitin disk (Fig. 5). Synthesis of 1,3-beta-linked glucans, which confer most of the rigidity to the yeast cell wall, is accounted for by the redundant 1,3-beta-glucan synthases Fks1 and Fks2, which in turn are effectors of the Rho1 GTPase (reviewed in [259]), while synthesis of mannoproteins requires a mannosyltransferase complex (reviewed in [260]. Formation of the secondary septum also involves directed secretion [199]. Strikingly, actin cables are oriented toward the bud neck and actin patches cluster at the bud neck during this process enforcing polarized vesicle traffic (Fig. 5). Finally, chitin synthase 3 (Chs3) also contributes to deposition of the secondary septum [261]. In contrast to deletion of *CHS2*, which abolishes primary septum formation and causes severe cytokinesis failure, deletion of *CHS3*, either alone or in combination with that of the third chitin synthase Chs1, does not cause obvious cytokinetic defects [251, 261, 262]. However, *chs2* mutant cells can survive thanks to the deposition of aberrant remedial septa mostly made by Chs3 that fill up the intercellular space [261, 263]. Remarkably, remedial septa are also built in the absence of CAR assembly, such as in *myo1∆* mutants, and therefore represent a major backup cytokinetic mechanism and a resource for cells to rapidly adapt to these adverse conditions [211].

Upon completion of a primary and secondary septum, the cell wall between mother and daughter cell is degraded by hydrolytic enzymes, such as the chitinase Cts1 [264] and several glucanases, including Dse4 and Egt2 [265, 266], thereby allowing cell separation (Fig. 5). Transcription of the genes responsible for cell wall digestion is driven by the Ace2 transcription factor and occurs only at the M to G1 transition of the cell cycle [267], thereby contributing to ensure proper timing of cell separation. Strikingly, the Ace2-dependent transcriptional program driving expression of most hydrolytic enzymes is restricted to the bud, thus explaining why after cell division a birth scar of undigested cell wall is only visible in the mother cells [15].

## The mitotic exit network

**Fig. 6:**
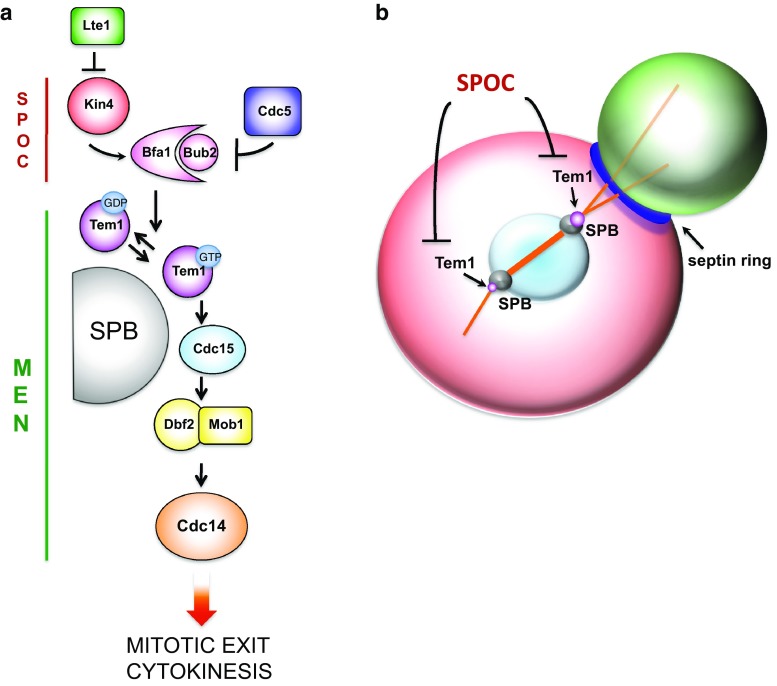
The mitotic exit network (MEN) and its regulation by the spindle position checkpoint (SPOC). **a** MEN signaling takes place mostly at SPBs, where the GTPase Tem1 in its active GTP-bound state promotes recruitment and activation of the Cdc15 protein kinase, which in turn recruits the Dbf2-Mob1 kinase complex that ultimately activates the Cdc14 phosphatase, thereby triggering mitotic exit and cytokinesis. Tem1 is kept inactive by the GTPase-activating protein Bfa1-Bub2 that can be inhibited by the polo kinase Cdc5, whose activity is counteracted by the kinase Kin4 in the mother cell. In turn, the Lte1 protein, which is localized specifically in the bud, restrains Kin4 in the mother compartment. **b** The MEN inhibitor Kin4 and the MEN activator Lte1 are spatially segregated in the mother and bud compartment, respectively (Kin4 *red*, Lte1 *green*). As long as an SPB has not moved into the bud, Tem1 and MEN are kept inactive, thereby coupling spindle positioning and nuclear division with mitotic exit

The budding yeast Mitotic Exit Network (MEN), is an essential kinase cascade that is similarly organized to the fission yeast Septation Initiation Network and the metazoan Hippo pathway (reviewed in [268–270]). MEN plays a crucial role in actin repolarization at the end of mitosis, as well as in cytokinesis, and comprises an upstream GTPase (Tem1), its effector Cdc15 kinase, the Mob1-Dbf2 kinase and the Cdc14 phosphatase. The polo kinase Cdc5 activates Tem1 by dampening the activity of the two component GTPase-activating protein Bub2-Bfa1, which keeps a large pool of Tem1 inactive until telophase (reviewed in [271]). Other upstream regulators, such as the polo kinase Cdc5, the bud-localized cortical protein Lte1 and the mother cell-specific Kin4 kinase, modulate Tem1 activation especially in relation to spindle positioning and nuclear division (Fig. 6a). Tem1 and MEN are indeed the targets of the Spindle Position Checkpoint (SPOC), which keeps Tem1 inhibited until the spindle elongates properly along the mother-bud polarity axis in anaphase, thereby preventing mitotic exit and cytokinesis in case of spindle misalignment (reviewed in [12, 13]; Fig. 6b). Intriguingly, proper mitochondrial inheritance from the mother to the daughter cells is required for the function of MEN in cytokinesis [272], suggesting that budding yeast cells keep MEN activity in standby until a balanced set of chromosomes and organelles have been segregated to the bud.

The core MEN actors are thought to work in a linear cascade (Tem1 > Cdc15 > Mob-Dbf2 > Cdc14), although feedback controls by Cdc14 on various MEN components have been discovered [273–275]. MEN signaling for mitotic exit (i.e., inactivation of mitotic CDKs) occurs at spindle pole bodies (SPBs, i.e., the budding yeast microtubule-organizing centers) by recruitment of MEN components to the SPB scaffold Nud1 (reviewed in [268, 276]; Fig. 6).

The Cdc14 phosphatase promotes mitotic exit in two ways, i.e., by inhibiting mitotic cyclinB-CDKs and by reversing CDK-driven phosphorylation events [277]. MEN is strictly required for its full activation in telophase (reviewed in [278]). Thus, one reason for MEN being involved in cytokinesis is linked to inactivation of CDKs, which otherwise prevent cytokinesis in many eukaryotic systems (reviewed in [8]). For instance, in budding yeast CDK inactivation in telophase is required for actin repolarization and targeted secretion at the bud neck [42, 120]. Many MEN proteins, however, relocalize from SPBs to the bud neck upon mitotic exit, suggesting the existence of additional direct roles in cytokinesis regulation [130, 279–285]. This is indeed the case. Although to date the list of MEN-regulated cytokinesis proteins is likely incomplete, we know several examples of cytokinesis factors that are phosphorylated by MEN kinases or dephosphorylated by Cdc14 (see below). Furthermore, inactivation of MEN proteins in conditions that allow mitotic exit prevents CAR contraction [161, 250]. It is interesting to note that the fission yeast Septation Initiation Network, while being similarly organized to the MEN and involving orthologous proteins, is specifically required for cytokinesis and dispensable for mitotic exit [268, 286].

Although MEN has been clearly involved in CAR constriction and cytokinesis (see below), its role in CAR assembly is controversial. Some MEN mutants have been reported to fail recruiting F actin to the CAR at restrictive temperature [35, 246, 280, 287], while others were shown to proficiently assemble an apparently functional actin ring [112, 130, 282]. Since activation of the Cdc14 phosphatase is required for actin ring formation [246], it is quite surprising that the upstream MEN factors are dispensable for this process. Likely, the ability of some MEN mutants to assemble a functional CAR is ascribable to an incomplete inactivation of the corresponding MEN proteins.

An important target of Cdc14 in CAR assembly is the IQGAP Iqg1 (see “The septin ring”). Iqg1 is phosphorylated in vivo by mitotic CDKs both in *S. cerevisiae* [288, 289] and in *C. albicans* [238] and is dephosphorylated by Cdc14 [246]. Mutating the CDK-dependent phosphorylation sites of Iqg1 to non-phosphorylatable alanines leads to premature CAR assembly before anaphase, thus recapitulating the phenotype of cells that either overexpress *CDC14* or have reduced levels of mitotic CDKs [238, 246, 290, 291]. Additionally, expression of non-phosphorylatable Iqg1 rescues the inability of *cdc14* mutant cells to assemble the F actin ring at restrictive temperature [246], suggesting that Iqg1 is a crucial MEN target in this process.

The chitin synthase Chs2 is phosphorylated by mitotic CDKs and dephosphorylated by Cdc14 to promote its timely relocalization from the ER to the bud neck [249]. Cdc14 has also been shown to interact with and dephosphorylate both formins Bni1 and Bnr1. In *cdc14* and *cdc15* mutants Bni1 fails to localize to the bud neck, whereas *CDC14* overexpression in metaphase displaces Bnr1 from the CAR while recruiting Bni1 [117]. Furthermore, Inn1 recruitment to the neck and activity is likely regulated by CDK-dependent phosphorylation and subsequent dephosphorylation mediated by Cdc14 [181, 250, 255], although the exact mechanism underlying this control remains to be defined. Finally, high CDK activity inhibits the daughter-specific transcriptional program responsible for the expression of the septum-degrading enzymes at the end of cytokinesis through phosphorylation of the transcription factor Ace2, while Cdc14 reverses inhibition [15, 16, 267, 292, 293]. Additional potential cytokinesis targets of mitotic CDKs and/or Cdc14 have been recently identified and hold promises for exciting discoveries in the future [117, 181].

Although activation of the Cdc14 phosphatase appears to be the main function of MEN in cytokinesis [181, 291], upstream MEN factors might contribute directly to this process. For instance, the MEN kinase Dbf2 directly phosphorylates Chs2 and likely stimulates its removal from the CAR by endocytosis [251]. Furthermore, Dbf2-dependent phosphorylation promotes activation of the Chs2 regulatory complex Hof1–Inn1–Cyk3 in several ways. On one hand, MEN contributes to the efficient recruitment of Chs2, Hof1, Inn1 and Cyk3 to the bud neck even independently of mitotic exit [250]. On the other, Hof1 phosphorylation by Dbf2 dissociates it from the septin ring and relocalizes it to the CAR [228].

## Concluding remarks

The last 20 years have witnessed a blooming of papers addressing the mechanisms that regulate cytokinesis. The budding yeast *S. cerevisiae* remains an outstanding model system to study this process, as many of the basic principles underlying cytokinesis are conserved in more complex eukaryotes.

In spite of cutting-edge technologies that have considerably improved the resolution of cytokinetic events and the enormous efforts by researchers in the field, many important questions await an answer, such as how precisely the CAR is organized and what contributes to its contraction, how formins promote CAR assembly, how CAR constriction is coupled to membrane addition, what drives the splitting of the septin ring, which are the critical targets of mitotic CDKs and MEN in cell division, and so forth.

One major obstacle to the progress of our knowledge in this field is the redundancy and intertwinings of cytokinetic pathways and proteins involved, which often makes the contribution of each hard to assess. Nevertheless, we can therefore expect in the years to come exciting discoveries that will shed light on such a fascinating and intricate process providing, hopefully, a complete and detailed picture of cytokinesis.
